# Extract from Broccoli Byproducts to Increase Fresh Filled Pasta Shelf Life

**DOI:** 10.3390/foods8120621

**Published:** 2019-11-27

**Authors:** Luisa Angiolillo, Sara Spinelli, Amalia Conte, Matteo Alessandro Del Nobile

**Affiliations:** Department of Agricultural Sciences, Food and Environment, University of Foggia, Via Napoli, 2571121 Foggia, Italy; luisa.angiolillo@unifg.it (L.A.); sara.spinelli@unifg.it (S.S.); matteo.delnobile@unifg.it (M.A.D.N.)

**Keywords:** shelf life, byproducts, fresh pasta, vegetable extracts, antimicrobial activity

## Abstract

The aim of the study was to evaluate the efficacy of extract from broccoli byproducts, as a green alternative to chemical preservation strategies for fresh filled pasta. In order to prove its effectiveness, three different percentages (10%, 15%, and 20% *v*/*w*) of extract were added to the filling of pasta. A shelf life test was carried out by monitoring microbiological and sensory quality. The content of phenolic compounds before and after in vitro digestion of pasta samples was also recorded. Results underlined that the addition of the natural extract helped to record a final shelf life of about 24 days, that was 18 days longer in respect to the control sample. Furthermore, results highlighted that the addition of byproducts extract to pasta also increased its phenolic content after in vitro digestion. Therefore, broccoli byproducts could be valorized for recording extracts that are able to prolong shelf life and increase the nutritional content of fresh filled pasta.

## 1. Introduction

Pasta is one of the main constituents of the Mediterranean diet as it contains significant amounts of complex carbohydrates, proteins, B-vitamins, and iron [1]. Pasta can be made with different kinds of flours (semolina, farina, wheat flour, etc.) mixed with water. Fresh pasta has more than 24% moisture and its water activity ranges from 0.92 to 0.99, thus it requires refrigeration [2]. It can be prepared with eggs in the dough or by filling a sheeted dough with a spiced mixture of ground meat, cheese or vegetables as for tortellini and ravioli. In the last decade, fresh filled pasta gained great national and international popularity, even though distribution beyond the Italian borders still represents a real problem due to the rapid microbial proliferation. In fact, this product is very susceptible to spoilage microorganisms and therefore, addition of preservatives or reduced oxygen packaging are necessary to prolong the shelf life that, even under refrigerated temperatures, lasts only two or three days [3]. Specifically, the pH of pasta without any preservatives may drop, thus indicating spoilage and increased coliforms [4]. Italian law [5] prescribes a pasteurization treatment before final packaging in order to reduce the growth of vegetative microbial forms and also to improve cooking behaviors. Generally, the thermal treatment is carried out in an injected steam belt pasteurizer and in addition to reducing water activity, it helps to increase starch gelatinization with consequently less water absorption during cooking [6]. For shelf life prolongation different methods have been applied to fresh pasta; the most common approach is based on chemical preservatives, as organic acids, and modified atmospheres (MAP) with low O_2_ concentrations (below atmospheric levels) and high CO_2_ concentrations (20% or higher), proper coupled with N_2_ as an inert gas filler [7,8,9,10]. Castelvetri et al. [11] demonstrated that packaging atmospheres with more than 30% CO_2_ were capable of extending fresh filled pasta shelf life, even if low residual O_2_ in the package headspace may cause mold growth. The search for more natural technologies of food preservation greatly promoted the exploration of antimicrobial compounds of vegetable or animal origin, as essential oils, enzymes or chitosan [12]. Among natural antimicrobial compounds, substances recordable from leaves, flowers, seeds, and peels are becoming very interesting [13]. Many components with useful properties can be found in food byproducts [14]. The possibility to recycle food byproducts may also represent a mean to face their environmental and economic impact. Among plants, *Brassica oleracea,* that belongs to the Brassicaceae family, represents one of the most abundant byproducts producers. It comprises 3500 species such as cauliflower, broccoli, kale, cabbage, and Brussels sprouts. Byproducts from *Brassica oleracea* are rich in phenols, as flavonoids, phenolic acids, and tannins, usually extracted by solvent extraction [15]. This extraction method has low selectivity and utilizes high energy cost, elevated solvents, and high temperatures [16]. For this reason, alternative methods of extraction have been investigated. Among them, the supercritical fluid extraction (SFE) with improved selectivity, automation, and environmental safety, represents a valid alternative [17].

According to the above-reported considerations, the aim of the study was to evaluate the efficacy of broccoli byproducts extract, obtained by SFE, to improve fresh filled pasta shelf life. To this aim, bioactive substances were first extracted from broccoli byproducts and subsequently added to fresh filled pasta to verify their effects on microbial and sensory quality. The evaluation of the consequent polyphenols content in pasta samples was also assessed.

## 2. Material and Methods

### 2.1. Raw Materials

Broccoli stems and leaves (*Brassica oleracea*) were provided by a local company in Foggia, Southern Italy. The samples were dried at 30–35 °C in a dryer (SG600, Namad, Rome, Italy) for 48 h. The dried samples were reduced to fine powder (≤250 µm) by a hammer mill (16/BV-Beccaria s.r.l., Cuneo, Italy) and then stored at 4 °C until further utilization.

### 2.2. SFE

Supercritical fluid extraction was carried out to collect active compounds from byproducts, above all polyphenolic compounds. It was performed using process conditions (150 bars, 35 °C, 20% ethanol and 10 min of dynamic extraction time) previously described by Arnáiz et al. [18], by the supercritical fluid extractor Speed SFE-2 (Applied Separation, Allentown, USA). The extract was placed overnight in vacuum oven at 30 °C to remove ethanol. The solid residue was collected in 25 mL of water.

### 2.3. Fresh Filled Pasta Production

Fresh pasta samples were produced with durum semolina (provided by Agostini mill Montefiore dell’Aso, Ascoli Piceno, Italy). Semolina and distilled water (30% *v*⁄*w*) were mixed for about 20 min to prepare the pasta dough. The samples were prepared using a pilot scale extruder (60VR; Namad, Rome, Italy) equipped with a roller sheeter (Raff, Minipan, Massa Lombarda, Italy) and a compressor (mod. Rondostar, Rondo Doge, Burgdorf, Switzerland) in order to obtain a 6 mm sheeted dough. The filling was prepared by mixing 65% (*w*/*w*) ricotta cheese, 19% (*w*/*w*) grated cheese, 16% (*w*/*w*) fresh spinach, 0.30% (*w*/*w*) potato flour, and 0.01% (*w*/*w*) salt. The sheeted dough and filling were combined in a modified double sheet ravioli machine (mod. PRP 300, Genoa, Italy) to prepare 12 cm diameter fresh pasta samples in the form of ravioli. Each sample consisted of two square dough sheets containing the filling. Four different formulations were prepared: fresh filled pasta without any addition (CNT), and the other three samples with increasing concentrations of broccoli extract in the filling: 10% (*v*/*w*) (10-BE); 15% (*v*/*w*) (15-BE), and 20% (*v*/*w*) (20-BE). The product was conveyed through a 3-m chamber equipped with a perforated steel conveyor belt (Custom, Italgi, Genoa, Italy). By steam injection at 91 ± 1 °C for 9 min, the pasteurization was carried out. After pasteurization the product passed through two fans to eliminate the condensed vapor on the surface and then was cooled to 4 °C and packaged in bags with anti-fog high-barrier multilayer film made up of polyethylene terephthalate, ethylene-vinyl alcohol, and polyethylene. The film oxygen transmission rate (OTR) was 6.19 cc/m^2^/day, the water vapor transmission rate (WVTR) was 1.208 g/m^2^/day, and the thickness was 50 μm (Di Mauro Officine Grafiche spa, Salerno, Italy). All the samples were stored for about 2 months at 4 °C, without light.

### 2.4. Microbiological Analyses

For microbiological analyses, about 10 g of sample were aseptically removed from each package, placed in a stomacher bag, diluted with 90 mL of sterile NaCl solution, and homogenized with a stomacher LAB Blender 400 (Pbi International, Milan, Italy). Serial dilutions in sterile saline solution were plated onto appropriate media. The media and conditions were the following: plate count agar (PCA) incubated at 30 °C for 48 h for aerobic mesophilic bacteria and at 7 °C for 10 days for psychrotrophic bacteria; Violet Red Bile Glucose Agar (VRBGA) incubated at 37 °C for 24 h for *Enterobacteriaceae*; Baird-Parker Agar, supplemented with egg yolk tellurite emulsion, incubated at 37 °C for 48 h for *Staphylococcus* spp.; Sabouraud Dextrose Agar, added with 0.1 g/L chloramphenicol (C. Erba, Milan, Italy), incubated at 25 °C for 48 h for yeasts and 25 °C for 5 days for molds. Reinforced Clostridial Medium (Oxoid, Milan, Italy) was used for the sulfite-reducing clostridia; after heat treatment of samples at 80 °C for 10 min to destroy the vegetative cells, the plates were incubated at 37 °C for 48 h in anaerobic conditions, thus avoiding contact with air. The count was carried out with the most probable number (MPN) method. Aerobic spore-forming bacteria were detected and counted on Nutrient Agar (Oxoid, Milan, Italy) after 48 h at 30 °C; all vegetative forms were previously destroyed by heat treatment of samples at 80 °C for 10 min. All media and supplements were from Oxoid (Milan, Italy). All microbiological analyses were performed twice on two different samples (one sample from two different trays). In order to quantitatively determine the microbial acceptability limit (MAL), a modified version of the Gompertz equation was fitted to the experimental data, as reported in previous studies [19,20]. The Italian law [5] fixes the threshold for total microbial count (TMC), staphylococci, and clostridia at maximum values of 10^6^, 5 × 10^3^, and 10^3^ Colony Forming Unit (CFU)/g, respectively.

### 2.5. Sensory Analysis

During the entire storage period, at selected times, both uncooked and cooked fresh pasta samples were subjected to a time intensity evaluation. Towards the aim, eight trained tasters were involved in the panel test. The panelists were asked to evaluate color, odor, and overall quality of uncooked samples and color, odor, taste, consistency, and overall quality of pasta cooked in food grade tap water at 100 °C. A nine-point rating scale, where 1 corresponded to ‘extremely unpleasant’ and 9 to ‘extremely pleasant’, was used to perform the panel test. [21]. The panelists were selected on the basis of their sensory skills (ability to accurately determine and communicate the sensory attributes, the appearance, odor, flavor, and texture). Prior to testing pasta, the panelists were trained in the sensory vocabulary and identification of particular attributes, by using commercial pasta. The analyses were performed in isolated booths, located in a standard taste panel kitchen. In order to determine the sensory acceptability limit (SAL), intended as the storage time to reach the sensory threshold, a modified version of the Gompertz equation was fitted to the sensory data [19,20]. The sensory threshold was set equal to 5.

### 2.6. Chemical Analyses

#### 2.6.1. Extraction of Polyphenols from Cooked Pasta Samples

The extraction of polyphenols from both control and enriched pasta samples was based on the method also described by Rashidinejad et al. [22]. Briefly, 1 g of each cooked sample was homogenized and extracted in a water bath with 50 mL of 95% methanol containing 1% HCl at 50 °C and 200 rpm. The mixture was cooled, filtered, and washed with 2 mL of the same solvent.

#### 2.6.2. In Vitro Digestion of Cooked Pasta Samples

Simulated gastric and intestinal digestions were carried out on both control and enriched pasta samples using the method of Rashidinejad et al. [22]. In brief, 1 g of each cooked sample was added with 10 mL of simulated filtered gastric fluid (SGF) at 37 °C and incubated in an orbital shaker at 37 °C at 235 rpm for 10 min. After adjusting the pH of the solution to 2.0, the treatment continued for a further 2 h at 95 rpm. Then, 36 mL at 37 °C of simulated intestinal fluid (SIF) were added to each gastric digestion sample and stirred at 37 °C at 95 rpm for 4 h. After 30 min from the beginning, the pH was adjusted to 6.8. In order to prepare the SGF sample, 2 g of NaCl, 7 mL of HCl (36%), and 3.2 g of purified porcine pepsin (in 1 L of deionized water, pH 1.2) were used, while to prepare the SIF sample a monobasic potassium phosphate solution (6.8 g in 250 mL of deionized water) with 77 mL of sodium hydroxide (0.2 M) and 500 mL of deionized water was mixed. Finally, 10 g of pancreatin and 0.05 g of porcine bile extract were added to the mixture and the pH was adjusted again to 6.8. For each sample, the digestion was carried out in triplicate.

#### 2.6.3. Total Phenolic Content

To measure the total phenolic content (TPC) in all the undigested and digested samples, the Folin–Ciocalteu assay was used. TPC was determined as described by da Silva et al. [23] with slight modifications. Briefly, 0.5 mL of sample (that obtained in Section 2.6.1 and that recorded in Section 2.6.2) and 2.5 mL of Folin–Ciocalteu reagent diluted in water (1:10 ratio) were left to rest for 5 min. An amount of 2 mL of Na_2_CO_3_ (4 g/100 mL) was then added. The mixture was allowed to rest again for 2 h in darkness. The absorbance was read by a spectrophotometer (UV1800, Shimadzu Italia s.r.l.) at 740 nm. Total phenols content was expressed as mg of gallic acid equivalents (GAEs) per g of pasta, according to a previously recorded calibration curve. For each sample, the analyses were carried out in triplicate.

### 2.7. Statistical Analysis

Experimental data were compared by one-way ANOVA analysis. A Duncan’s multiple range test, with the option of homogeneous groups (*p* < 0.05), was used to determine significance among differences. To this aim, Statistica 7.1 for Windows 152 (StatSoft Inc., Tulsa, OK, USA) was used.

## 3. Results and Discussion

### 3.1. Total Phenolic Content

TPC of both undigested and digested pasta samples is shown in Table 1. As can be seen, the TPC in the indigested control samples (0.63 mg GAEs/g) was significantly (*p* < 0.05) lower than that found in pasta enriched with broccoli extract (1.84–1.86 mg GAEs/g), even if it did not increase linearly with the quantity of added extract. This finding may have been due to the interaction of polyphenols with ricotta proteins and the concomitant formation of less active complexes [24]. Gallo et al. [25] also stated that milk protein fractions caused a decrease of the in vitro antioxidant activity of polyphenols, as a consequence of the weaker non-covalent bonds between proteins and polyphenols.

The other information that can be deduced looking at Table 1 is that the phenolic content of the digested samples appear to be higher in respect to the indigested ones, with the highest value found in the 20-BE sample (2.61 mg GAEs/g).

The explanation for this trend could be linked to the hydrolyzation exerted by digestive enzymes towards chemical bonds in the phenolic-protein complexes, promoting in this way a greater release and extractability of phenolics at the in vitro level [26,27]. In particular, Gumienna et al. [28] claimed that the action of gastric-digestive enzymes may lead to the development of aglycones phenolic compounds, more reactive than the corresponding glycoside forms. These findings positively encourage the consumption of pasta enriched with broccoli byproducts extract, as a way to promote intake of valuable food for human health, since it is widely demonstrated that phenolic compounds provide extraordinary anticancer, antiviral, antibacterial, cardio-protective, and anti-mutagenic activities [29].

### 3.2. Quality of Fresh Filled Pasta

**Figure 1:**
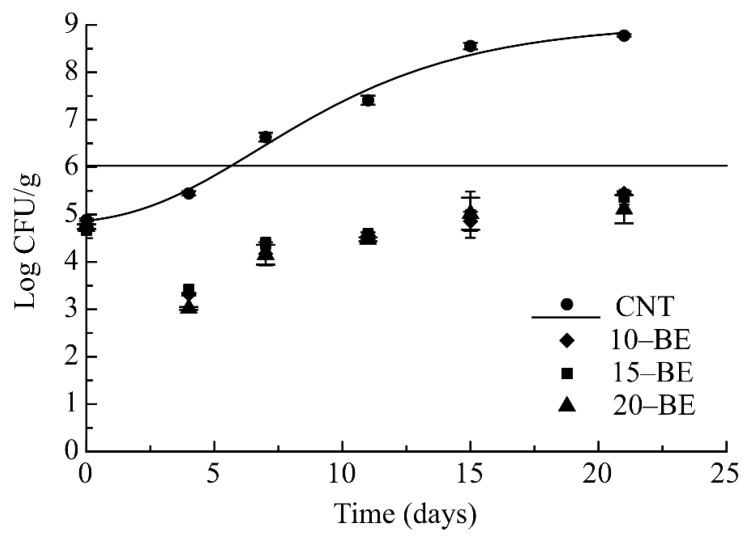
Evolution of mesophilic bacteria in pasta samples during storage at 4 °C. CFU: Colony Forming Unit.

Figure 1 describes the growth of total mesophilic bacteria in all the experimental samples. Table 2 reports values of fitting parameters. As can be inferred from the data, in the CNT pasta an immediate growth with an ascendant trend was found, with values from 4.88 log CFU/g to 8.77 log CFU/g until the 22nd day.

In the control pasta the microbial acceptability limit was reached after six days. On the contrary, the three samples with increasing concentration of broccoli extract, revealed a positive microbial quality during the entire observation period, without significant difference among them. These data were similar to the microbial trend of psychrotrophic bacteria. 

**Figure 2:**
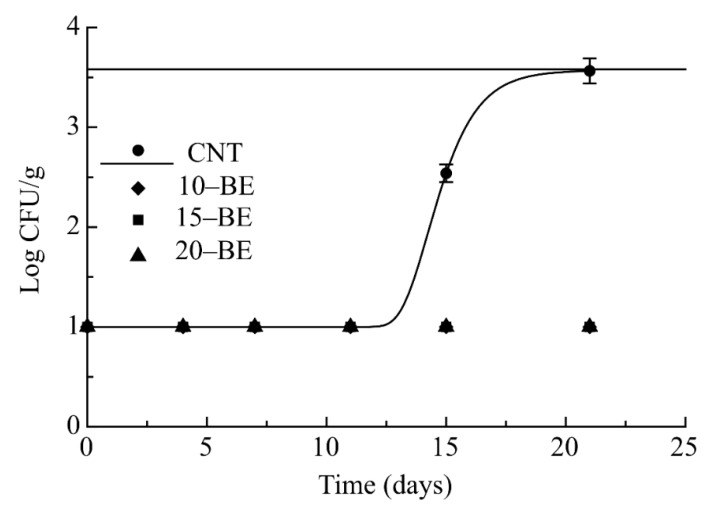
Evolution of *Staphilococcus* spp. in pasta samples during storage at 4 °C.

Regarding *Staphylococcus* spp., counts were found below the microbial limit for the entire 25 days of observation in all the active pasta samples, while in the CNT microbial growth started from the 15th day and reached the limit after 20 days of storage (Figure 2). Data of MAL are also reported in Table 2.

The addition of broccoli extract appeared to be also effective against *Enterobacteriacae*, molds, and yeasts as these microbial groups in the enriched pasta did not grow until the 25th day (data not shown). On the contrary, the CNT sample revealed molds growth, both visible and on plates, at the 13th day (Table 2), *Enterobacteriacae* and yeasts around 5.75 log CFU/g and 5.59 log CFU/g after 20 days. The trend of control samples is not surprising because it is in accordance with other studies on fresh filled pasta [10,11,12]. Clostridia and aerobic spore forming bacteria were never found in any samples (data not shown).

The results of the microbial quality confirm findings of other authors about the antimicrobial properties of vegetal extracts [30,31,32], even though the studies were all carried out under in vitro conditions. The application carried out in the current study, not only assessed the potential effects of extract from broccoli byproducts, but also demonstrated that it is possible to significantly extend microbial stability (Table 2). These data appeared to be of particular importance since the literature highlighted that fresh filled pasta packaged in ordinary atmosphere and stored at 4 °C generally reached very short shelf life values, accounting for hours to one week, depending on the hygienic production conditions [12]. Another import consideration is that the antimicrobial effect is not determined by the quantity of extract added to the experimental samples. As a fact, looking at Table 1, polyphenols appeared to be similar among samples with broccoli extract. It has been suggested by different authors that the bioactivity of polyphenols is rather related to their structure than to their quantity, with differences from one polyphenol to another [33,34,35]. Their structure is also dependent on temperature and, in particular, heat treatment [36].

According to the above-mentioned results, the microbiological acceptance of pasta was limited to about six days of storage for the CNT sample and lasted about 24 days for the active samples (Table 2).

Sensory evaluation was carried out for more than one month to know when the product became unacceptable for undesired sensory changes. Specifically, color evaluation allowed recording different results among samples, depending on the extract concentration added to the filling, because extract addition modified the filling color from a whitish green of the CNT to an intense green of the 10-BE and 15-BE ravioli samples, to a too dark green of the 20-BE pasta sample. Therefore, while the first two active samples were accepted for a long period, the 20-BE sample was refused within about two weeks.

Regarding pasta consistency, a gradual hardening of all the samples was found, an inevitable consequence of both pasteurization and storage. As described by [11] pasteurization may influence the texture of fresh filled pasta. In fact, during the thermal treatment hardening takes place due to a different distribution of water within the matrix. The increase in water–starch bonding results in a decrease of available water for the other components of the matrix, thus influencing protein denaturation and starch gelatinization. The addition of the extract helps to obtain a more hydrated structure, in fact, the CNT sample revealed a harder consistence in respect to the other three samples. The consistence of the 10-BE sample was found better than that of 15-BE and 20-BE samples because these two types of ravioli revealed an excessive liquid consistence of the filling. The taste of enriched pasta was perceived acceptable by the panelists, even if they underlined increasing the quantity of broccoli extract, the bitterness of the product increased.

**Figure 3:**
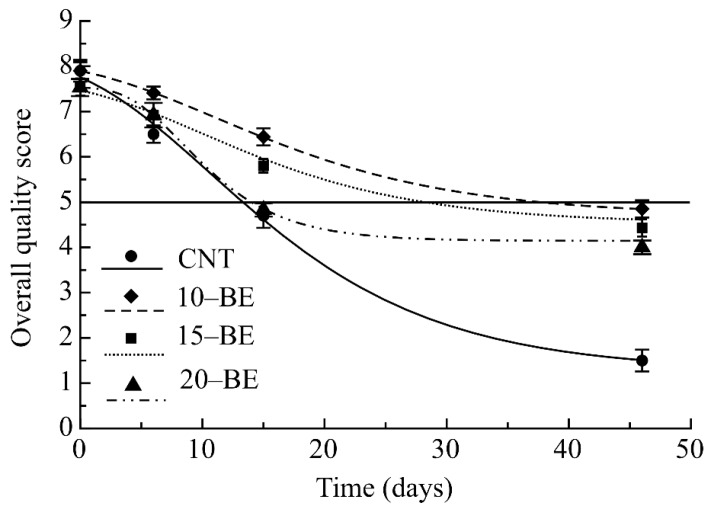
Sensory quality of uncooked fresh filled pasta during storage at 4 °C.

The overall quality of both uncooked and cooked samples reflected the above discussed trends of specific sensory parameters without great differences between uncooked and cooked products. As an example, Figure 3 reports the trend of overall quality of uncooked pasta. All SAL values are reported in Table 2. It is evident looking at Figure 3 and at the sensory data of Table 2 that the CNT sample appeared to be the less appreciated, being rejected after about 13 days, due to undesired changes in color and consistence. The 20-BE samples were refused within 15 days of storage above all for the undesired color, 15-BE samples remained acceptable for less than one month when uncooked and more than one month when considered after cooking, whereas the 10-BE samples recorded the highest score before and after cooking, with sensory acceptability accounting for more than 40 days.

Taking into account both MAL and SAL values of Table 2, the shelf life was reported as the lowest value among them. From the results it is possible to highlight that excessive proliferation of mesophilic bacteria provoked in most cases the end of the product shelf life, whereas in the case of the pasta with the highest concentration of extract (20-BE) the main problem was the presence of unacceptable sensory defects. The effects of the extract were very tangible because while the CNT sample was refused after a few days, the two active samples 10-Be and 15-BE remained acceptable for more than 20 days because the extract controlled microbial proliferation and delayed undesired sensory changes.

## 4. Conclusions

Reuse of food byproducts in a sustainable way may be a possible way to reduce environmental impact and face costs related to their disposal. In this study extract from broccoli byproducts was added for the first time to the filling of fresh pasta to improve quality and prolong shelf life. Results underlined that the addition of broccoli extract helped to record a final shelf life of about 24 days, that was 18 days longer in respect to the control sample. In addition, pasta with broccoli extract showed a higher phenolic content in respect to the free samples, particularly after digestion. The most appropriate extract amount was 10%, with the pasta samples appreciated for color, taste, and consistency.

## Figures and Tables

**Table 1:** Total phenolic content (TPC) of undigested and digested cooked ravioli samples.

	TPC (mg GAEs/g Ravioli)	
	Undigested	Digested
CNT	0.63 ± 0.03 ^a^	0.81 ± 0.01 ^a^
10-BE	1.84 ± 0.08 ^b^	2.13 ± 0.10 ^b^
15-BE	1.84 ± 0.18 ^b^	2.23 ± 0.10 ^b^
20-BE	1.86 ± 0.12 ^b^	2.61 ± 0.16 ^c^

^a–c^ Data in columns with different letters are significantly different (*p* < 0.05). GAEs: gallic acid equivalents; CNT = fresh filled pasta without any addition; 10-BE = fresh filled pasta with 10% broccoli extract; 15-BE = fresh filled pasta with 15% broccoli extract; 20-BE = fresh filled pasta with 20% of broccoli extract.

**Table 2:** Shelf life (day) of pasta samples during storage at 4 °C, calculated as the lowest value between microbial acceptability limit (MAL) and sensory acceptability limit (SAL) (mean ± SD).

Sample	Microbial Quality (Day)			Sensory Quality Uncooked Pasta (Day)	Sensory Quality Cooked Pasta (Day)	Shelf Life (Day)
	MAL ^Mesoph^	MAL ^Staph^	VMT	SAL	SAL	
CNT	5.57 ± 0.33 ^a^	22.4 ± 0.10	13	12.95 ± 0.26 ^a^	12.45 ± 0.10 ^a^	5.57 ± 0.33 ^a^
10-BE	24.21 ± 0.11 ^b^	˃25	˃25	40.91 ± 0.23 ^d^	43.25 ± 0.20 ^d^	24.21 ± 0.11 ^c^
15-BE	23.72 ± 0.12 ^b^	˃25	˃25	27.76 ± 0.15 ^c^	39.16 ± 0.15 ^c^	23.72 ± 0.12 ^c^
20-BE	24.33 ± 0.10 ^b^	˃25	˃25	14.54 ± 0.21 ^b^	19.10 ± 0.17 ^b^	14.54 ± 0.21 ^b^

^a–d^ Data in columns with different letters are significantly different (*p* < 0.05). VMT = visible molds time (day); CNT = fresh filled pasta without any addition; 10-BE = fresh filled pasta with 10% broccoli extract; 15-BE = fresh filled pasta with 15% broccoli extract; 20-BE = fresh filled pasta with 20% of broccoli extract.
